# Droplet-Based Microfluidic High Throughput Screening of *Corynebacterium glutamicum* for Efficient Heterologous Protein Production and Secretion

**DOI:** 10.3389/fbioe.2021.668513

**Published:** 2021-05-07

**Authors:** Suvasini Balasubramanian, Jun Chen, Vinoth Wigneswaran, Claus Heiner Bang-Berthelsen, Peter Ruhdal Jensen

**Affiliations:** ^1^Research Group for Microbial Biotechnology and Biorefining, National Food Institute, Technical University of Denmark, Kongens Lyngby, Denmark; ^2^Samplix ApS, Herlev, Denmark

**Keywords:** droplet-based microfluidics, high throughput screening, heterologous protein production, β-glucosidase, α-amylase, *Corynebacterium glutamicum*

## Abstract

With emerging interests in heterologous production of proteins such as antibodies, growth factors, nanobodies, high-quality protein food ingredients, etc. the demand for efficient production hosts increases. *Corynebacterium glutamicum* is an attractive industrial host with great secretion capacity to produce therapeutics. It lacks extracellular protease and endotoxin activities and easily achieves high cell density. Therefore, this study focuses on improving protein production and secretion in *C. glutamicum* with the use of droplet-based microfluidic (DBM) high throughput screening. A library of *C. glutamicum* secreting β-glucosidase was generated using chemical mutagenesis coupled with DBM screening of 200,000 mutants in just 20 min. Among 100 recovered mutants, 16 mutants exhibited enhanced enzyme secretion capacity, 13 of which had unique mutation profiles. Whole-genome analysis showed that approximately 50–150 SNVs had occurred on the chromosome per mutant. Functional enrichment analysis of genes with non-synonymous mutations showed overrepresentation of genes involved in protein synthesis and secretion relevant biological processes, such as DNA and ribosome RNA synthesis, protein secretion and energy turnover. Two mutants JCMT1 and JCMT8 exhibited the highest secretion with a six and a fivefold increase in the β-glucosidase activity in the supernatant, respectively, relative to the reference strain JC0190. After plasmid curing, a new plasmid with the gene encoding α-amylase was cloned into these two mutants. The new strains SB024 and SB025 also exhibited a five and a sixfold increase in α-amylase activity in the supernatant, respectively, relative to the reference strain SB023. The results demonstrate how DBM screening can serve as a powerful development tool to improve cell factories for the production and secretion of heterologous proteins.

## Introduction

Genetic engineering of microbes has diverse applications in biotechnology, enabling improved and valuable products for human consumption, industrial applications and therapeutic solutions. Significant advances have been made from rational gene over-expression and the introduction of heterologous genes with fine control of gene expression and modulating the regulatory networks of the host cell (Jensen and Hammer, 1998; Solem and Jensen, 2002). Over the years, the integration of systems biology and synthetic biology has allowed for the development of high performing cell factories. The production of proteins and chemicals have proven to be economical and environmentally sustainable by endowing desirable properties on microorganisms by rerouting existing metabolic pathways or introducing entirely new ones (Lee et al., 2012). In order to exploit cell factories for efficient heterologous expression, optimization of the properties of the strains and enabling secretion of proteins into the culture media at high efficiency are important. It is also critical to understand the complex molecular mechanisms of the cells in order to fully harness the technology in delivering the desired phenotypes and products.

Challenges remain in rational engineering of hosts for improved protein production and secretion, as protein synthesis is a complex machinery, and multi-level cellular processes must be well coordinated to achieve high-yield production and secretion. Incorporating traditional techniques like random mutagenesis and screening is again becoming an alternative to rational engineering. This technique involves introducing random mutations into the genome of the cell to construct a cell library with diverse phenotypic variations followed by screening for a desired phenotype. Due to the random nature of mutagenesis, a high throughput screening technology capable of screening about 10^6^–10^8^ mutants is required for screening a large cell library efficiently (Derkx et al., 2014).

Droplet-based microfluidics (DBM) is an emerging high-throughput technology with a lot of potential for screening large sets of mutant libraries individually (Chen et al., 2017). The platform generates compartments of volumes as low as femtolitres and perform reactions individually at the single cell level enabling rapid screening (Seemann et al., 2012). Monodisperse water-in-oil droplets (media containing cells enclosed by oil) are generated with tunable size at a high frequency in a microfluidic chip. These compartments of single-cell in a droplet increase the cell density in post-incubation and enable accumulation of the secretion in droplets, which are then screened individually at the frequency of 0.1–1 kHz (Theberge et al., 2010). All the reagents and equipment used for droplets screening are biocompatible and therefore target cells with improved performance can be recovered for further use.

*C. glutamicum* is an attractive candidate for the expression and secretion of heterologous proteins into the culture media enabling simpler downstream processing. This gram-positive soil bacterium is already prevalent as an industrial workhorse for generating essential biomolecules with applications in food, feed and pharmaceutical products such as vitamins and amino acids (Izumi et al., 1978; Krömer et al., 2005). *C. glutamicum* has several advantages such as GRAS (generally recognized as safe) and low nutrient requirements. It also lacks extracellular proteases, providing an oxidative environment for proper folding, and disulfide bond formation over the widely used production host *E. coli* (Berlec and Štrukelj, 2013) and has both twin arginine transporter (TAT) and secretion (SEC) pathways to transport the proteins across the cytoplasmic membrane (Liu et al., 2017).

Previous work has facilitated improved traits in the organism including over-expression tools and higher biomass to improve its potential as an industrial host for recombinant protein production (Kikuchi et al., 2009; Unthan et al., 2014). In this study, we used random mutagenesis and DBM high throughput screening to successfully improve heterologous protein production and secretion in *C. glutamicum.* The study demonstrated that DBM screening served as a high-throughput and cost-effective toolbox for improving protein production and secretion in Gram-positive bacteria. The results of the study will also facilitate reverse engineering strategies for further developing cell factories based on the valuable mutations identified.

## Results and Discussion

### Random Mutagenesis and Microfluidic Screening

**FIGURE 1:**
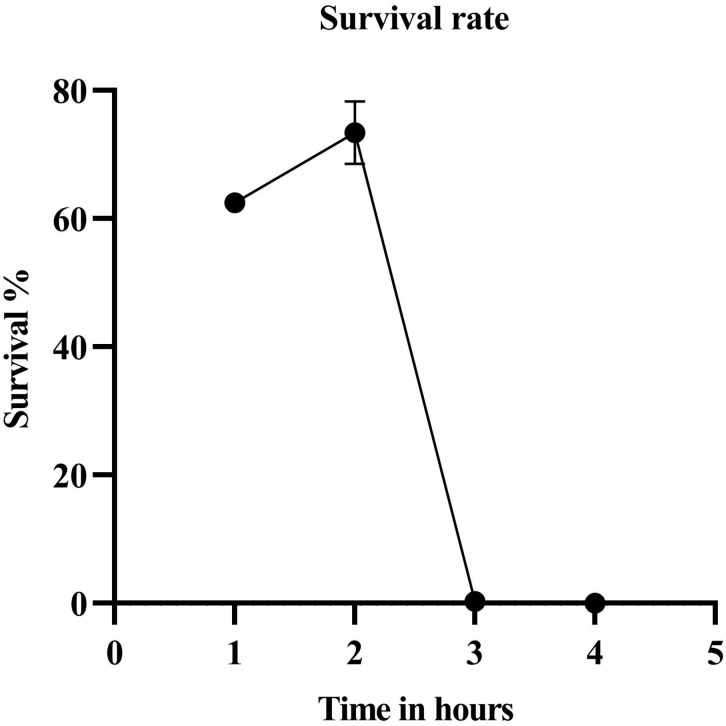
Survival rate after EMS treatment. The survival percentage of the cells by the end of the third hour was 0.315.

**FIGURE 2:**
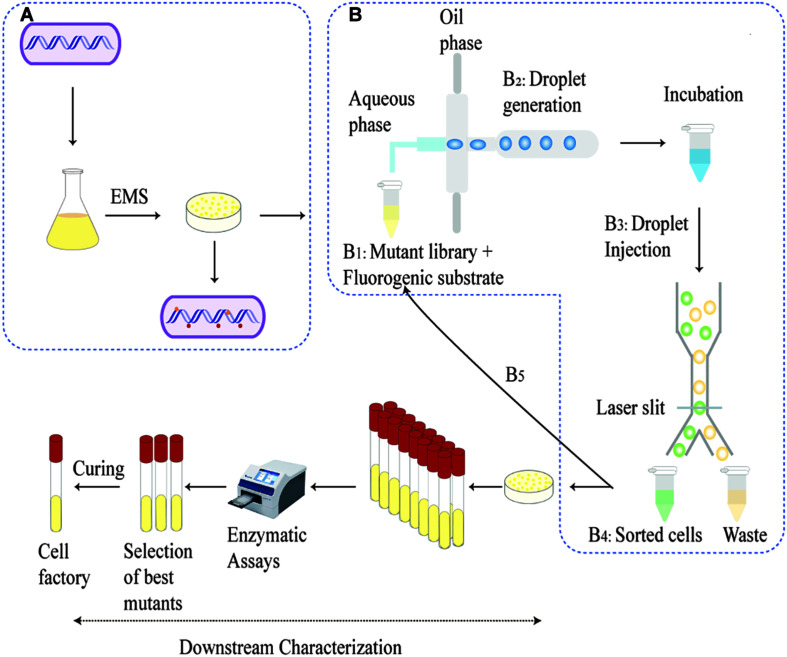
Schematic overview of development and screening of the mutant library: A whole genome mutated library was generated with EMS chemical mutagenesis on shake flask culture and were recovered on an agar as shown in **(A)**. Steps **B_1_–B_5_** represents the microfluidic process—The recovered mutants were diluted to 10^7^ mutant libraries mixed with fluorogenic substrate **(B_1_)**. The droplets generated incubates for the cells to accumulate secretion **(B_2_)**. Post incubation, droplets are injected into the sorting chip and sorted based on the fluorescence intensity and collected in an Eppendorf tube. The sorted cells are then released and injected into the device for a second round of droplet generation and screening **(B_5_)**. The sorted cells are then released and cultivated on agar plates. The outgrown single colonies are picked for downstream characterization, e.g., shake flask fermentation and protein secretion tests.

The parent strain JC0190 secreting β-glucosidase was mutagenized with ethyl methanesulfonate (EMS) and by the end of a 3-h treatment, 0.32% of the cells survived (Figure 1). We chose the library after 3-h EMS mutagenesis for the further experiment, as an approximately 99% killing rate would be eligible for droplet screening (Chen et al., 2017). Encapsulation of mutants in droplets was carried out along with the fluorogenic substrate Fluorescein Di-β-DGlucopyranoside (FDGlu) (Figure 2). The calculation of cell density for encapsulation utilizes the Poisson distribution, where the ratio of having single cells to multiple cells can be optimized and controlled by varying the cell density (Beneyton et al., 2017). In this study, a λ of 0.5 was used to generate an average of 0.5 cells per droplet. More than 200 K droplets were generated because of high single cell to multi-cell ratio will lead to a large fraction of empty droplets (Chen et al., 2018). The droplets were then incubated for 3–4 h, where the cells could proliferate and secrete β-glucosidase into droplets to hydrolyze the substrate, after which fluorescein was released to generate the green fluorescence for detection. Sorting of the mutants occurs based on the threshold set for the intensity of the fluorescent signals. 3.2% of the droplets with the highest signals were sorted from a screening of 140 K positive droplets in total in 20 min. The sorted droplets were collected in a 1.5-mL Eppendorf tube. The cells were then released and were re-injected into the device for the second round of encapsulation and sorting to further improve the purity. The second round of sorting yielded 3,900 sorted droplets (1.95%) resulting from screening a library of 200 K positive droplets.

**FIGURE 3:**
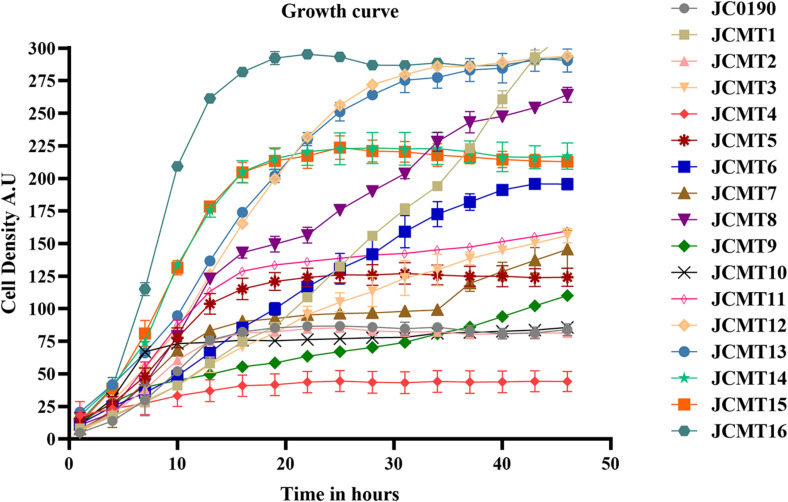
Growth curves of the parent and mutant strains carried out in BioLector. The growth of the strains were monitored in a 48-well flower plate (culture volume: 1,000 μL, temperature: 30∘C, agitation: 800 rpm) by measuring scattered light (ex: 620 nm, Gain: 20).

After being released from the sorted droplets, the cells were recovered on agar plates. Among the formed single colonies, 100 colonies were randomly picked and printed on a BHI agar plate containing IPTG, esculin and FeCl_3_ for indication of β-glucosidase activity. β-glucosidase cleaves esculin to produce esculetin and glucose molecule. The hydrolytic product esculetin reduces the ferric ions in the medium to produce iron, which causes browning on the agar. Therefore, the colonies producing brown color on the agar are esculin positive and possess β-glucosidase activity, and the shade of brown color could indicate the capacity of protein secretion, which served as an effective secondary screening (Veena et al., 2011). Sixteen colonies exhibiting the strongest dark red color were picked for further characterization. The growth of the strains was analyzed in microplates on Biolector (Figure 3).

### Characterization of the Selected Mutants

**FIGURE 4:**
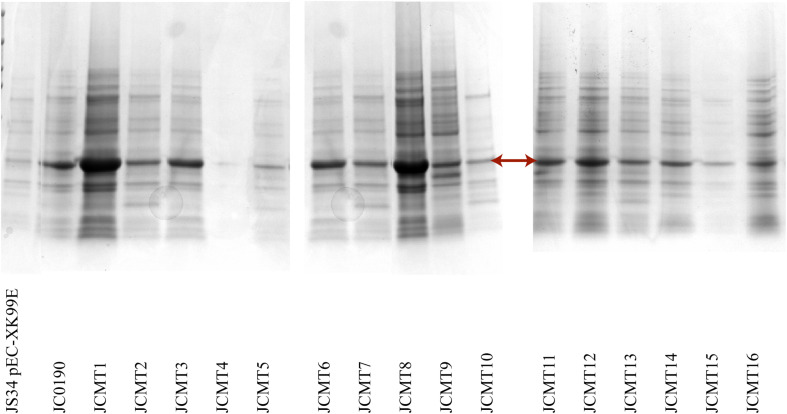
SDS PAGE analysis of the supernatant of the selected mutants. The arrow points the band β-glucosidase. The lanes from left to right are the wild-type strain with empty plasmid, and the reference strain followed by the mutants. 15 μL of acetone precipitated protein samples were loaded on the wells.

**FIGURE 5:**
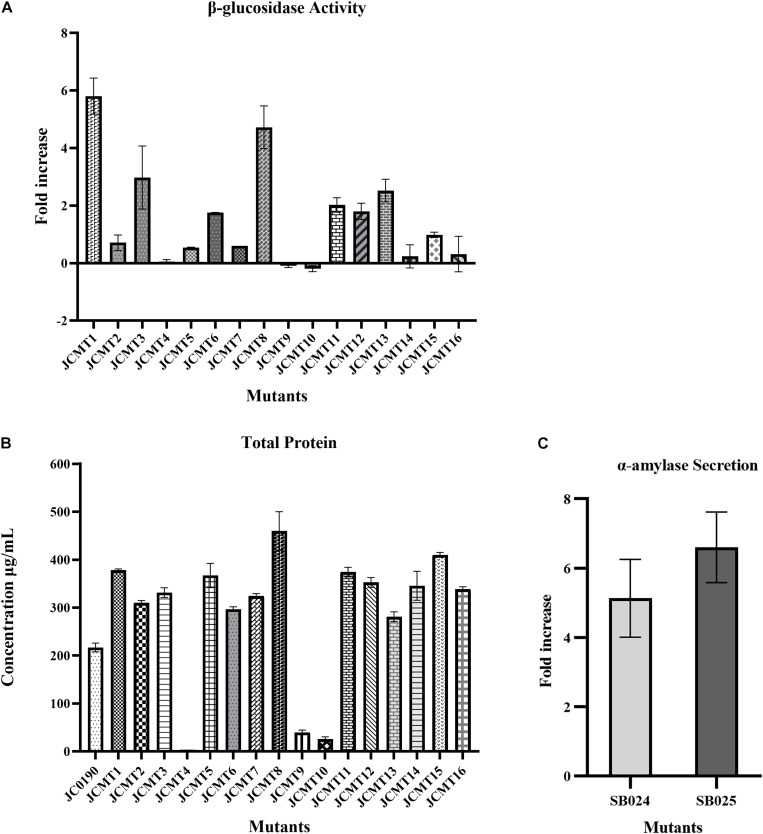
Protein secretion capacity of selected mutant strains: **(A)** β-glucosidase activity: fold increase in the relative fluorescence activity of the mutant strains secreting β-glucosidase in comparison to the reference strain JC0190. **(B)** Total protein: total protein content of the reference and mutant strains in the supernatants. The protein content of mutant 4 is around 4 μg and hence negligible in the graph. **(C)** α-Amylase secretion: fold increase in the α-amylase enzyme units of the strains SB024 and SB025 derived from mutants JCMT1 and JCMT8, respectively, compared to the reference strain SB023.

The β-glucosidase secretion capacity of the selected mutants was validated by testing the activity in the supernatant of the cultures. Thirteen of sixteen mutants had higher β-glucosidase activity in the supernatants compared to the reference strain JC0190. The supernatants were concentrated and analyzed on an SDS-PAGE gel, which showed improved protein secretion in the mutants. Although a native protein band overlaps with the β-glucosidase band on the SDS-PAGE gel in Figure 4 the protein concentration of the band from the strain JC0190 supernatant is higher relative to the negative control proving the presence of β-glucosidase. Based on the growth profiles generated by Biolector shown in Figure 3, 13 mutants were fast-growing strains with higher biomass and the strains JCMT02, JCMT09, JCMT10 had a similar profile compared to the reference strain JC0190. Amongst the mutant strains, JCMT08 had the highest biomass production. By analyzing the supernatant, the mutant JCMT1 and JCMT8 had the highest activity of β-glucosidase and total protein content in the supernatant (Figures 5A,B), where approximately six and fivefold higher enzyme activity, respectively, than the reference strain JC0190 were observed. However, JCMT8 had higher total protein content in the supernatant than JCMT1 (Figure 5B).

As JCMT1 and JCMT8 showed a better performance than the parent strain and the other mutants, they could potentially serve as platform strains for producing other proteins in *C. glutamicum*. To test this, the plasmid in JCMT1 and JCMT8 were cured using bactericidal antibiotic penicillin. The successful plasmid curing was confirmed by a growth inhibition in the presence of kanamycin and lack of β-glucosidase in the supernatant. The plasmid-cured mutants were transformed with a new plasmid with α-amylase gene (pECXCαAmy), which resulted in the strains SB024 and SB025 derived from JCMT1 and JCMT8, respectively. The new strains SB024 and SB025 harboring the gene coding for α-amylase exhibited similar growth rate as that of the uncured counterparts (Supplementary Figure 1) and had a 5.6 and 7-fold higher secretion of α-amylase compared to the reference strain SB023 (JS034 harboring pECXCαAmy), respectively (Figure 5C). These results demonstrates both the mutant’s stability and its potential for use as a host for producing other essential proteins. With mutagenesis and DBM screening experiment, we have thus established *C. glutamicum* strains with up to sixfold higher activity in the supernatant, which is comparable to the previous work with other organisms such as yeasts, where the enzyme activity was improved between two and fourfold by mutagenesis and DBM screening (Huang et al., 2015; Beneyton et al., 2017).

### Genome Resequencing and SNV Identification

The genomes of all the 16 mutants and the parent strain JC0190 were sequenced in an attempt to disclose the link between genetic and phenotypic changes. Single nucleotide variants (SNVs) of the 16 mutants were called and filtered against the SNVs identified in JC0190 to eliminate background noises. It was found that the mutants JCMT03, JCMT06, JCMT12, and JCMT13 had the identical SNV profiles, which indicates that they originated from the same mutant, and were repetitively picked during the secondary selection on agar plates. Therefore, JCMT03 was used to represent the four strains in the subsequent analysis.

**FIGURE 6:**
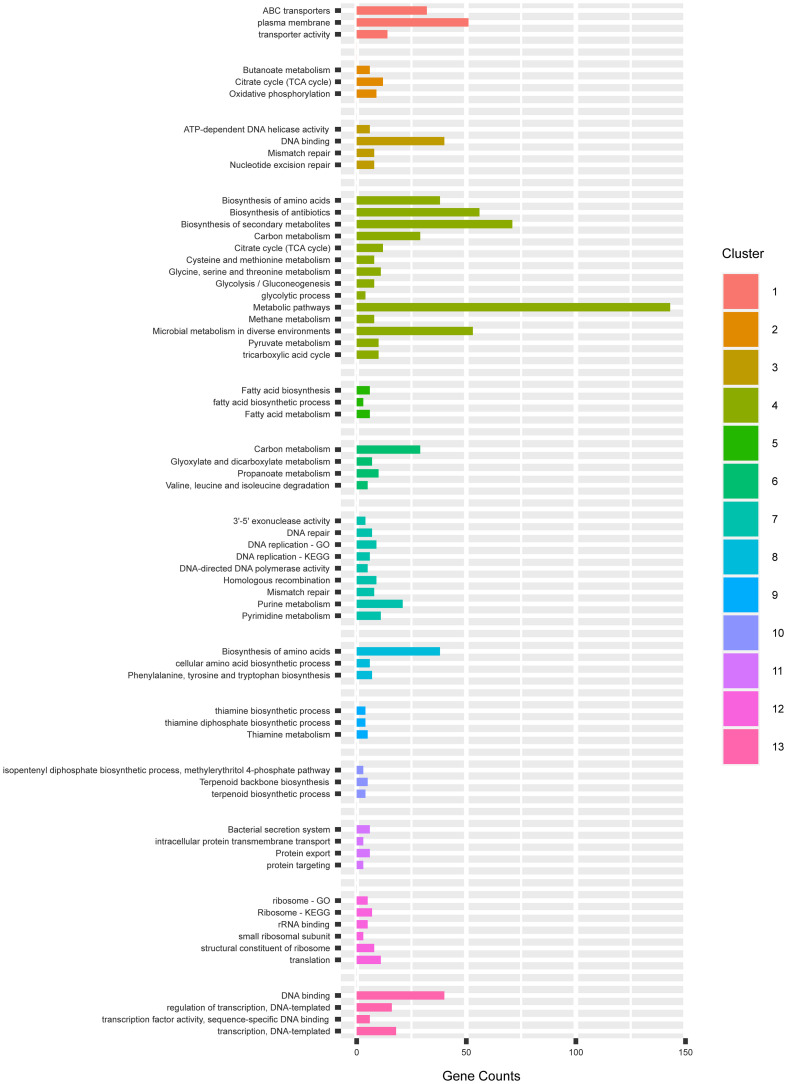
The gene clusters enriching mutations in GO and KEGG categories by DAVID.

In the 13 mutants with unique SNV profiles, we found approximately 50–150 SNVs per genome, and 0–1 SNV on the expression plasmid in each strain. The occurrence of SNVs on the genome and the plasmids were thus 0.002–0.005% and 0–0.012%, which were not significantly biased. In total, 1,002 mutations were identified across 13 mutants of which 313 were silent mutations, 566 were missense and 123 were in the non-coding region. To explore the patterns of these mutations, we first pooled all the genes that had non-synonymous SNVs in the coding regions in the 13 mutants and submitted this pool, which had 713 unique gene entries, to DAVID (Huang et al., 2009) for functional enrichment and clustering. The GO and KEGG terms were chosen for the analysis, which resulted in 13 functional clusters (Figure 6).

In the cluster 1, 3 terms in relation to membrane transport activity were enriched. They mostly consisted of different ABC transporter permeases and cation transporters. In the plasma membranes, the subunits b, c and γ of the ATP synthase F_0_F_1_ were enriched. The F_0_F_1_ synthase protein complex consisting of the subunits α_3_, β_3_, γ_1_, δ_1_, ε_1_, a_1_, b_2_, and c_*n*_ (n—varies from species to species) present on the bacterial membranes are responsible for the synthesis of ATP from ADP, being a key parameter of energy production of aerobic cells (Soga et al., 2017). The α_3_, β_3_, γ_1_, δ_1_, ε_1_ subunits catalyzes ATP hydrolysis, while the a_1_, b_2_, and c_*n*_ mediates proton transport across the membrane (Ueno et al., 2005). In *E. coli*, the deletion of the ATP operon encoding ATPase has resulted in decreased ATP synthesis despite the increased respiration rate (Jensen and Michelsen, 1992), which resulted in decreased growth rate (74–78%) and yield (55–58%) than the wild type strains. The mutants JCMT1, JCMT10, JCMT14 had missense mutations on the subunits γ, b, and c of ATP synthase, respectively, while the mutant JCMT16 had a silent mutation on the γ subunit, which indicates the capacity of ATP synthesis could be altered in the mutants in favor of protein synthesis.

In the cluster oxidative phosphorylation, the three subunits of the ATP synthase F_0_F_1_ in the cluster 1 reoccurred. In the term plasma membrane, we also found non-synonymous SNVs also existed in the genes *secA2*, *secD* and *secF* in the sec pathway. *SecA* is responsible for transferring of secretory proteins to the membrane-embedded translocon, and *SecD* and *F* are responsible for releasing the mature peptides into periplasm (Chatzi et al., 2014). The mutations on *SecA* were enriched in mutants 11 and 16 while the mutation on *SecD* is only in 11. However, in this study, a tat signal peptide was used for mediating protein secretion, and hence the effect of this mutation on secretion is unclear.

**FIGURE 7:**
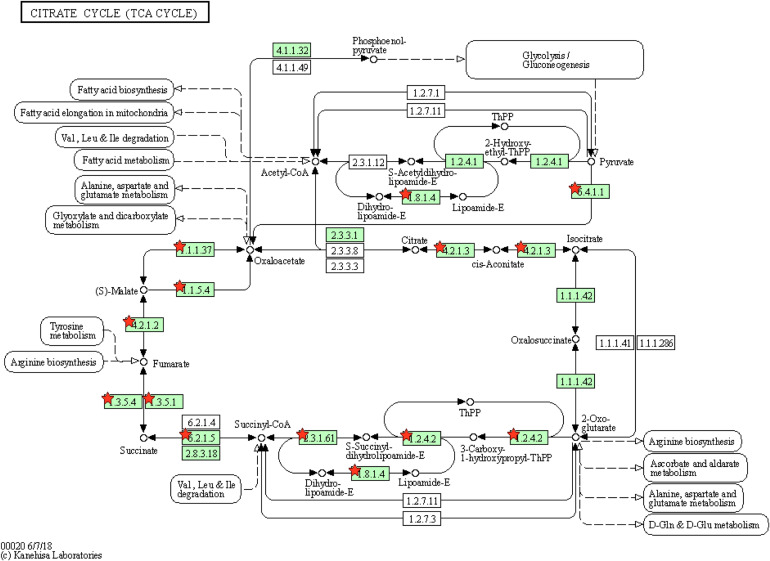
Tricarboxylic acid cycle with genes enriching mutations. The red stars represent the genes with presence of mutations.

In the cluster 2, we found two interesting terms, namely, TCA cycle and oxidative phosphorylation. Non-synonymous SNVs were found in 12 genes in the TCA cycle e.g., pyruvate carboxylase (*pyc*), acetate (*ace*), fumarase (*fum*) and succinic dehydrogenase (*sdh*) (Figure 7). The TCA cycle provides several metabolic precursors and cofactors for cell growth and amino acid production. The pyruvate OAA node is one of the factors regulating the carbon flux in the TCA cycle by interconnecting different metabolic pathways responsible for carbon metabolism. The pyruvate carboxylase (*pyc*) which is part of the anaplerotic pathway enhances the cell growth and amino acid production on glucose by renewing OAA, and the expression levels of *pyc*, *ace*, *fum* and *sdh* are enhanced with biotin supplementation (Xu et al., 2018). The mutants JCMT7, JCMT10, and JCMT14 possessed a mutation in the *pyc* gene, where the mutants JCMT7 and JCMT10 had a non-synonymous mutation with R/C and P/L substitutions while JCMT14 had a synonymous mutation. Similarly, three mutants—JCMT1, JCMT8 and JCMT11 had SNV mutations in the *sdh* gene, of which JCMT1 had a non-synonymous mutation with S/L substitution and the rest had silent mutations. The high number of mutations in the genes involved in the primary metabolic pathways such as TCA cycle indicated an enhanced metabolic activity could be critical for protein synthesis and secretion.

Moreover, three genes encoding cytochrome b subunit of the bc complex, cytochrome bd-type quinol oxidase, subunit 1 and 2, respectively, which are involved in the respiratory chain were found in cluster 2. The efficiency of the respiratory chain depends on the expression levels of the bc1 complex and the cytochrome bd oxidase. The bc1-aa3 branch is important for aerobic growth in minimal media such as CGXII while the cytochrome bd oxidase inhibits the growth, biomass and the protein production levels when overexpressed (Bott and Niebisch, 2003). The S/L mutation in the cytochrome bd-type quinol oxidase, subunit 1 could have positively influenced the capacity of respiration in the strain JCMT1.

In the cluster 3, it mostly consists of terms that have relations to DNA replication and repair. Forty genes were allocated in term DNA binding. Among others, we found several genes that encode DNA gyrases, DNA polymerases, helicases and regulators.

The cluster 4 is the biggest group containing 14 terms. These terms mostly consist of primary and secondary metabolisms, in which the TCA cycle occurred in cluster 2. Furthermore, we found 8 genes involved in the glycolysis pathway. In cluster 5, we found that 6 genes in the fatty acid synthesis had non-synonymous SNVs. Considering the fatty acid synthesis pathway is composed of 9 genes in total. The frequency of mutations was high in the fatty acid biosynthesis pathway. The cluster 6, 8, 9, and 10 consist of genes involved in primary and secondary metabolism, which also occurred in cluster 4.

The cluster 7 resembles the cluster 3 involved in DNA replication and repair, but the cluster 7 had more detailed classification. In addition, we also found that 21 genes were involved in purine metabolism, and 11 genes were involved in pyrimidine metabolism. In the cluster 11, it is interesting to see that 6 genes involved in protein secretion were mutated. These genes encode accessory Sec system translocase SecA2, inner membrane protein translocase YidC, preprotein translocase subunit SecD, preprotein translocase subunit SecF, preprotein translocase subunit YajC, signal recognition particle GTPase.

The cluster 12 contains terms involved in translation and ribosome process. We saw mutations in several genes that encode ribosome 30s and 50s subunits, which play a critical role in translation. Terms in the cluster 13 were mostly involved in the regulation of transcription, in which transcriptional regulators were clustered.

For the mutations on the plasmid, in the 13 unique mutants, we found a single SNV on the expression plasmid in the strain JCMT01, 03, 05, and 08. In JCMT16, there were two SNVs on the plasmid. In JCMT01 and JCMT03, the SNV is non-synonymous and changed the coding sequence of the kanamycin resistance gene. In JCMT05, the SNV was also located in the kanamycin resistance gene, but it was synonymous. The rest were in the intergenic regions. The mutation on the resistance gene or on the plasmid is not critical. As by plasmid curing and introducing the newly constructed plasmid expressing α-amylase, the high protein production and secretion traits remained in the mutants, indicating that the improvement was a result of the mutations on the chromosome rather than those on the plasmid (Figure 5C).

## Conclusion

DBM has previously been explored to screen mutants generated using random mutagenesis for optimization of strains producing native and non-native metabolites (Huang et al., 2015; Chen et al., 2018). Results from the current study has shown how one can improve phenotypic traits of *C. glutamicum* for increasing protein secretion using high-throughput droplet screening. Furthermore, the diversity of mutations profiles within a single mutant disclosed in this study gives multiple possibilities for future metabolic engineering aimed at improving protein secretion.

## Materials and Methods

### Strains and Plasmids

In this study, *C. glutamicum* JS34, a derivative of ATCC 13032, Shen et al. (2017) was used as the host for strain construction. The vectors used for the construction of expression/secretion plasmid were pEC-XK99E and pEC-XC99E harboring kanamycin and chloramphenicol resistance, respectively. For construction, the β-glucosidase expression/secretion plasmid (pECXKBGL), the SD sequence of the *tpiA* gene (30 bp before the start codon) from *C. glutamicum* followed by Cgr0949 signal peptide and the β-glucosidase gene from *S. degradans* were cloned onto the *Xba*I site on pEC-XK99E, and the plasmid was transformed into JS34, which resulted in JC0190. JCMT1 to JCMT16 are the mutants derived from JC0190 after mutagenesis and DBM screening. For the construction of the amylase expression/secretion strains, the mutant strains JCMT1 and JCMT8 were cured and transformed with the plasmid pECXCαAmy containing α-amylase gene from *G. stearothermophilus*, named SB024 and SB025, respectively. Table 1 summarizes the strains and plasmids used in this study.

**TABLE 1:** Summary of strains and plasmids used.

**Strains and plasmids**	**Description**	**References**
JS34	A derivative of the wild-type strain ATCC 13032 carrying an *attB* site in its chromosome	Shen et al., 2017
JC0190	JS34 strain expressing and secreting β-glucosidase	This work
JCMT1–JCMT16	Mutant derivatives of JC0190	This work
SB023	JS34 strain expressing and secreting α-amylase	This work
SB024	Cured JCMT1 expressing and secreting α-amylase	This work
SB025	Cured JCMT8 expressing and secreting α-amylase	This work
pEC-XK99E	Cloning vector Km^*r*^, *Ptrc* promoter, *lacIq* regulatory gene	Kirchner and Tauch, 2003
pEC-XC99E	Cloning vector Cm^*r*^, *Ptrc* promoter, *lacIq* regulatory gene	Kirchner and Tauch, 2003
pECXKBGL	pEC-XK99E vector containing the SD sequence of the *tpiA* gene, signal peptide Cg0949, and gene coding β-glucosidase	This work
pECXCαAmy	pEC-XC99E vector containing the SD sequence of the *tpiA* gene, signal peptide Cg0949, and gene coding α-amylase gene	This work

### Media and Culture Conditions

For all fermentations, CGXII media supplemented with 0.1% brain heart infusion broth (BHI, Sigma, St. Louis, MO, United States) was used (Unthan et al., 2014). The media composition per liter is 1 g BHI, 20g (NH_4_)_2_SO_4_, 1gK_2_HPO_4_, 1g KH_2_PO_4_, 5g urea, 13.25mg CaCl_2_⋅2H_2_O, 0.25g MgSO_4_⋅7H_2_O, 10mg FeSO_4_⋅7H_2_O, 10mg MnSO_4_⋅H_2_O, 0.02mg NiCl_2_⋅6H_2_O, 0.313mg CuSO_4_⋅5H_2_O, 1mg ZnSO_4_⋅7H_2_O, 42 g MOPS, 0.2mg biotin, 30 mg protocatechuic acid and 40g D-glucose. For the preparation of electro-competent cells, the standard procedure in the Handbook of *C. glutamicum* was followed (Eggeling and Bott, 2005). For β-glucosidase hydrolysis in the droplet, 50 μM FDGlu substrate from Invitrogen, CA, United States was used (Hardiman et al., 2010). The antibiotic concentrations were 25 μg/mL and 8 μg/mL for kanamycin and chloramphenicol, respectively. The Isopropyl ß-D-1-thiogalactopyranoside (IPTG, Sigma-Aldrich, St. Louis, MO, United States) concentration for induction was 1 mM. Ethyl methanesulfonate (EMS) from Sigma-Aldrich, St. Louis, MO, United States was used for chemical mutagenesis at a concentration of 2%. For indication of amylase activity on agar plates, 5 g/L starch, 15/L g agar, 1.5 g/L iodine and 1.5 g/L potassium iodide were added to the CGXII/BHI medium. All strains were grown at 30∘C aerobically for 24 h.

### *C. glutamicum* Mutant Library Construction

A single colony of the *C. glutamicum* construct JC0190 was inoculated into 10-ml BHI broth and cultivated 30 ml test tube with 220 RPM shaking at 30∘C overnight. The outgrown culture was first washed in PBS buffer (pH = 7.4) (Sigma-Aldrich, St. Louis, MO, United States) twice and resuspended in PBS, in which the cell density was adjusted to 10^9^ cells per mL. EMS was spiked into the culture at a concentration of 2%. It was incubated for 4 h at room temperature and sampled at a 1-h interval. Meanwhile, a tube with 10^9^/mL JC0190 cells without EMS was also incubated and sampled for calculation of the killing rate. During post mutagenesis, the samples were washed twice with CGXII—0.1% BHI to remove residual EMS. Both treated and untreated samples were plated on BHI agar plates with appropriate dilutions for counting CFUs and calculating the killing rate.

### Microfluidic Chip Fabrication

**FIGURE 8:**
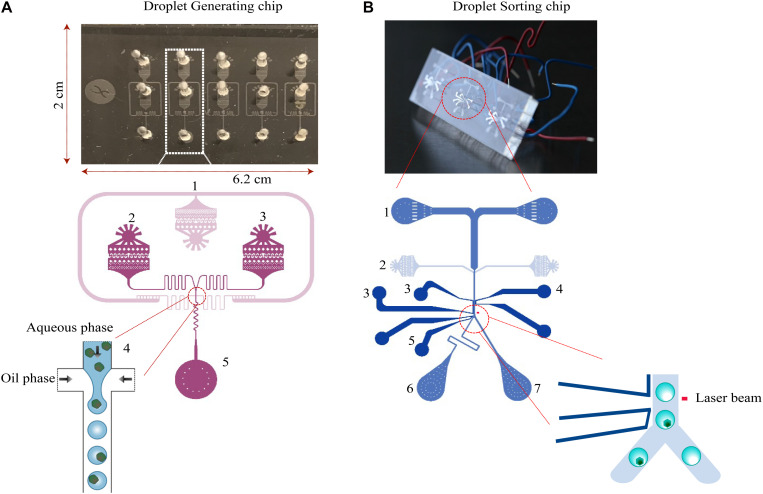
Design of microfluidic chips. **(A)** The droplet generation chip for cell encapsulation consists of an oil phase inlet (1), and two aqueous phase inlets (2 and 3). The fluid resistors (4) dampen fluctuations arising from the mechanical instability of syringe pumps and PDMS device. Due to the surface tension of the water–oil interface, the aqueous phase breaks up into droplets at the flow-focusing junction. Droplets are collected into a syringe or an Eppendorf tube from the outlet (5). **(B)** The droplet-sorting chip consists of two inlets for droplets (1) and spacing oil (2). The flow rate ratio of the oil and injected emulsions are varied to control the distance between adjacent droplets. The droplets at the sorting junction are sorted based on the fluorescence intensity. Downstream to the detection, electrodes—ground (3, 4), high voltage (5) are located. Next to the electrodes, the channel is split into two outlets for collecting sorted droplets (6) and unsorted/waste cells (7).

The two chips used for encapsulation and sorting of the droplets (Figure 8) are both made of structured polydimethylsiloxane (PDMS) slab bonded to a glass slide, which was fabricated by Droplet Genomics (Vilnius, 10223 Lithuania). The procedure is briefly described as follows: the 10:1 w/w mixture of PDMS (Sylgard 184, Dow Corning) is cast over a mold, created using regular UV-photolithography to pattern a layer of SU-8 2075 (MicroChem) on a 4-inch silicon wafer. The PDMS was cured overnight at 60∘C, cut into separate devices and carefully removed from the mold. The molded chips are transparent with a dimension of 6.2 × 2 × 0.5 cm. Holes for in- and outlets were made using Ø:0.75 mm biopsy punches. Both glass slides and PDMS parts were exposed to a 50 W 13.56 MHz air plasma for 60 s (Atto Plasma cleaner, Diener), then immediately bonded and placed under the weight (400 g/slide) at 90∘C for 10 min. After cooling down, the channels were flushed with filtered Aquapel and subsequently quickly purged by applying vacuum to the outlets. The sorting chips were heated on an 85∘C hotplate, allowing a low-temperature solder wire (Indalloy #19, Indium Corp) to be inserted and fill the electrode cavities. Before cooling the device, small pieces of wire were inserted as connectors to the electrodes. The design of droplet generation and sorting chip from Mazutis et al. (2013) were used in this study.

### Experimental Setup of Microfluidic Devices

The setup of microfluidic devices published in our previous studies was used (Chen et al., 2017). The system described here again in brief. A custom-built fluorescence microscope, with a 488-nm laser (06-01; Cobolt, Solna, Sweden) guiding through a 1,000-mm cylindrical lens (LJ1516RM-A; Thorlabs, Newton, NJ, United States) and a 10× microscope objective (N10X-PF; Thorlabs) was used to form a narrow line for fluorescence detection. The chips are placed on a 3D printed microscope stage. The fluorescent light emitted was transmitted through a dichroic mirror (MD499; Thorlabs) and a bandpass filter (MF530-43; Thorlabs) before being measured by a photomultiplier tube (PMT) (PMTSS; Thorlabs). A field-programmable gate array (FPGA) (PCIe-7842R; National Instruments, Austin, United States) was programmed to sample the PMT signal every 7 μs (>143 kHz) and continuously evaluate both the signal width and intensity. When a signal fell within the preset gating, the on-chip electrodes are activated with a square voltage wave (15 kHz) amplified to ±400 V (Trek 623B; Trek, Inc., Lockport, NY, United States). For droplet generation, the fluidic inlets/outlet were connected to a vertically oriented syringe pump (PHD 2000; Harvard Apparatus, Inc., Holliston, MA, United States) using PTFE tubing (TW30; Adtech, Stroud, United Kingdom) with an internal diameter of 320 μm. For sorting, fluorinated ethylene propylene (FEP) tubing (JR-T-6794-M10; VICI-Jour, Schekon, Switzerland) with an inner diameter of 100 μm was used instead for the fluidic outlets to ensure that the sorted drops were immediately pushed out of the tubing and collected into Eppendorf tubes. The controlling software package consists of two main parts: a LabVIEW FPGA code that is downloaded to the external processor (for fast and reliable operation) and a regular LabVIEW code (Supplementary File) supplying the user interface, which runs on the laboratory PC.

### Encapsulation of Mutants in Droplets

The library was diluted in CGXII media containing kanamycin, IPTG and FDGlu to get a final concentration of 10^7^ cells/ml. The culture, which forms the aqueous phase, and the oil-containing surfactant (Dolomite Pico-Surf 2.5%; Royston, United Kingdom) was set to 10 μL/min inflow rates to result in a droplet diameter of 50 μm (Chen et al., 2017). A 1-ml syringe (BD Medical, Sandy, UT, United States) was connected to the outlet to collect the emulsions and incubated for 3–4 h at 25∘C. Post incubation, the droplet emulsion containing the cells were examined on a microscope (Supplementary Figure 2). For the second round of screening, the sorted cells from the first round were reinjected for encapsulation followed by collection and incubation.

### Sorting the Cells Encapsulated in Microdroplets

The syringe containing the droplet emulsion were reinjected into the sorting chip at a rate of 1 μl/min, and the oil phase at 10 μl/min. The cells were sorted based on the fluorescence intensity of the preset threshold level; when the cells with fluorescence exceeding the threshold pass, an electric field is activated by supplying a voltage to the on-chip electrodes. The threshold is set to sort roughly 2–3% of droplets with the highest fluorescent readouts. For each sorting operation, the electrical field was triggered for 1,750 μs after a 250-μs delay to allow droplets to pass the gap between the laser spot and the electrodes.

### Extraction of Cells After Sorting

The collected emulsions were centrifuged at 100 g for 30 s. The oil layer was carefully removed and 300 μl of PFOH (1*H*,1*H*,2*H*,2*H* perfluorooctan-1-ol) (Sigma-Aldrich, St. Louis, MO, United States) was added to disrupt the droplets and release the cells into the aqueous layer. The cells were then plated onto BHI agar plates to form colonies.

### BioLector Cultivations

Strains were cultured in a 48 well flower plate (m2p-labs GmbH, Baesweiler, Germany) with 1 mM IPTG concentration and 25 μg/mL kanamycin at 30∘C with continuous shaking at 800 rpm and the increase of biomass was monitored through BioLector (m2p-labs GmbH, Baesweiler, Germany). The humidity and gain were set to 95% and 20, respectively.

### Fermentation in Shake Flasks

For further large-scale analysis, fermentations were carried in 250 mL baffled shake flasks with a culture volume of 25 mL with 220-RPM shaking at 30∘C for 24 h. Upon completion, the cultures were centrifuged at 6,000 g for 30 min at 4∘C and the supernatants were collected for further analysis.

### Plasmid Curing

Plasmid curing is the process of eliminating the production plasmid from the hosts. From the overnight cultures of JCMT1 and JCMT8, a subculture was cultivated under selective pressure. During the log phase, penicillin (Sigma-Aldrich, St. Louis, MO, United States) at a concentration of 100 μg/mL was added (Tomás and Kay, 1984). During their decline phase, the cells were plated on a non-selective BHI agar and incubated for 24 h at 30∘C. Post incubation, the colonies were replicated on a new BHI agar plate with kanamycin and incubated for 24 h at 30∘C to identify the colonies without the production plasmid.

### Qualitative and Quantitative Assays

#### Enzyme Activity Measurement

The activity of β-glucosidase was measured by the hydrolysis of FDGlu at 525 nm. Cellulose from *T. reesei* (Sigma-Aldrich, St. Louis, MO, United States) was used as a standard. For α-amylase activity, ceralpha kit (Megazyme, Bray, Ireland) was used with α-amylase from *Aspergillus oryzae* (Sigma-Aldrich, St. Louis, MO, United States) as a standard.

#### Bradford Assay

Total protein concentration was measured using the Bradford assay. The Bradford reagent (Sigma-Aldrich, St. Louis, MO, United States) and samples were mixed to a ratio of 30:1. The samples were vortexed, incubated for 15 min and measured using a UV spectrophotometer at 595 nm. A standard calibration curve was plot using five concentrations of Bovine Serum Albumin (BSA, Sigma-Aldrich, St. Louis, MO, United States) 0.125, 0.25, 0.5, 1, and 1.5 mg/mL. The supernatant of JS34 bearing pEC-XK99E was used as a blank.

#### SDS PAGE

The supernatants from the fermentations were treated with acetone (Sigma-Aldrich, St. Louis, MO, United States) at −20∘C to precipitate the proteins. The protein pellets after centrifugation at 5,000 g for 10 min, were boiled with 4× laemmli buffer (Bio-Rad Laboratories, Hercules, California, United States) at 95∘C for 5 min. The samples were then loaded on a gradient (4–20%) mini-PROTEAN stain-free TGX precast polyacrylamide gels (Bio-Rad Laboratories, Hercules, California, United States) and analyzed on a Bio-Rad imager after electrophoresis.

### Genome Sequencing Analysis

Genomic DNA was purified from the mutant using DNeasy blood and tissue kit (Qiagen, Hilden, Germany), and the quality was assessed using DNA electrophoresis and a NanoDrop 1,000 spectrophotometer (Thermo Fisher Scientific, Waltham, MA, United States). Library preparation and sequencing were provided by BGI China (Shenzhen, China). The sequencing was performed on a BGIseq 500 next-generation sequencer, from which data sets (paired) per sample were obtained. Afterwards, adaptor sequences, contaminants, and low-quality reads were removed from the raw reads. CLC Genomics Workbench (Qiagen, Hilden, Germany) was used to map the reads, detect single-nucleotide variations (SNVs) and insertion/deletion events (INDELs), and identify genomic rearrangements using the published genomic sequence of *C. glutamicum* ATCC13032 (Kalinowski et al., 2003). To explore the patterns of mutations, all the genes that had non-synonymous SNVs in the coding regions in the 13 mutants were pooled and submitted to DAVID (Huang et al., 2009) for functional enrichment and clustering. The GO and KEGG terms were chosen for the analysis. All the sequencing data are deposited in the NCBI SRA database under the accession number PRJNA682818.

## Data Availability Statement

The datasets presented in this study can be found in online repositories. The names of the repository/repositories and accession number(s) can be found below: https://www.ncbi.nlm.nih.gov/, PRJNA682818.

## Author Contributions

JC designed and performed the experiments. SB performed the experiments and wrote the manuscript. All authors discussed the results and contributed to the final manuscript.

## Conflict of Interest

The authors declare that the research was conducted in the absence of any commercial or financial relationships that could be construed as a potential conflict of interest.

## Supplementary Material

Supplementary Figure 1 Growth curves of cured and uncured mutants. The growth of the strains were monitored in a 48-well flower plate (culture volume: 1000 μL, temperature: 30°C, agitation: 800 rpm) by measuring scattered light (ex: 620 nm, Gain: 20) on biolector.

Supplementary Figure 2 Microscopic view of droplet emulsion containing cells.
