# Supplementary figures and images for: Droplet-Based Microfluidic High Throughput Screening of Corynebacterium glutamicum for Efficient Heterologous Protein Production and Secretion
Source: Front Bioeng Biotechnol. 2021 May 7;9:668513. doi: 10.3389/fbioe.2021.668513 (PMC8137953; doi:10.3389/fbioe.2021.668513)

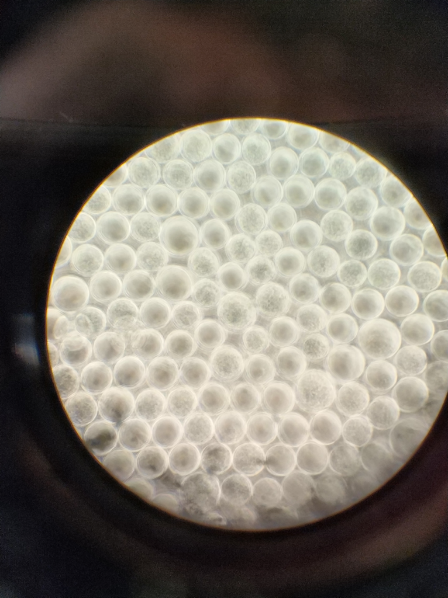

Supplement: Supplementary Figure 1 — Growth curves of cured and uncured mutants. The growth of the strains were monitored in a 48-well flower plate (culture volume: 1000 μL, temperature: 30°C, agitation: 800 rpm) by measuring scattered light (ex: 620 nm, Gain: 20) on biolector. [file Image_1.TIF]

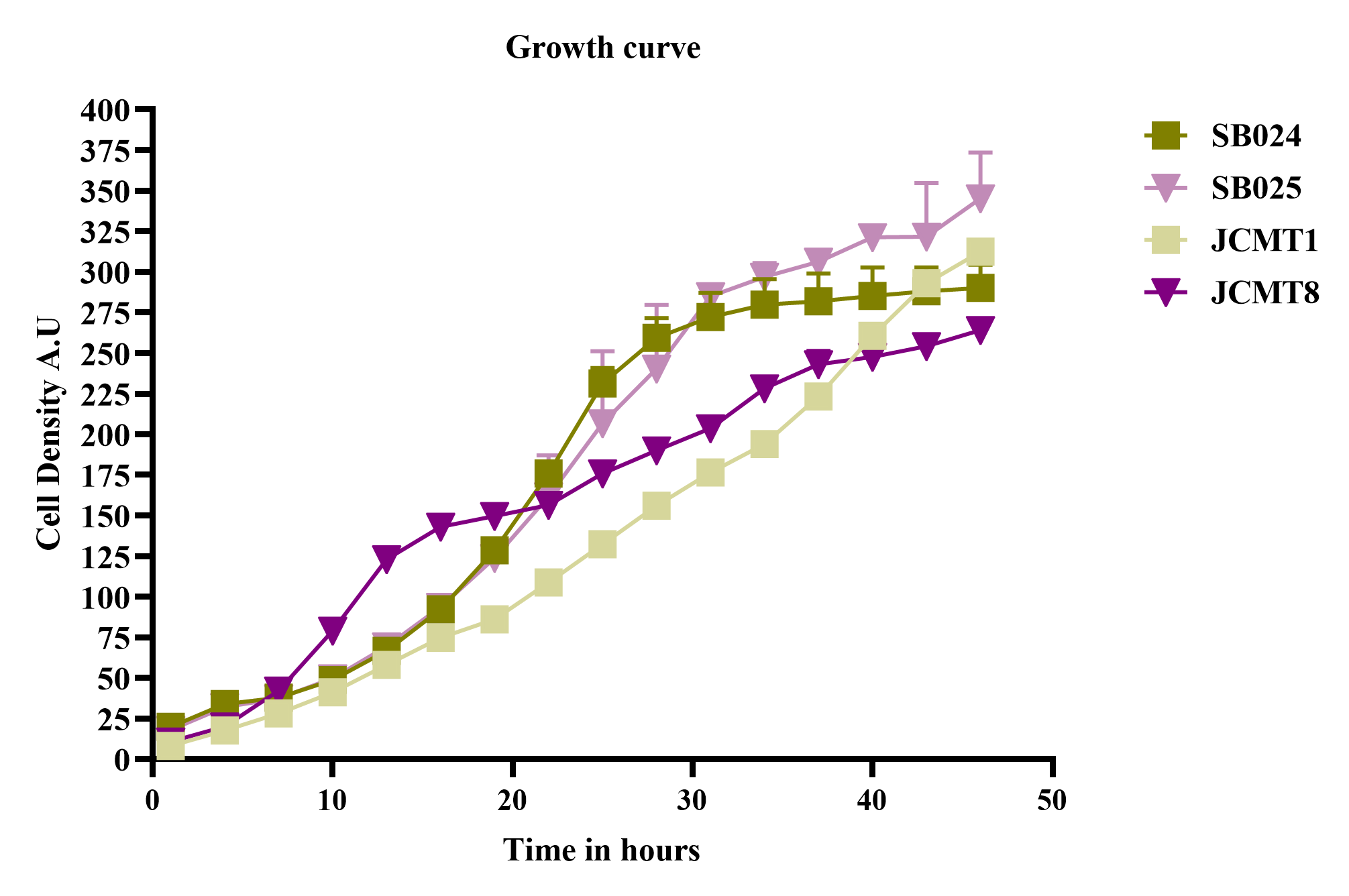

Supplement: Supplementary Figure 2 — Microscopic view of droplet emulsion containing cells. [file Image_2.TIF]

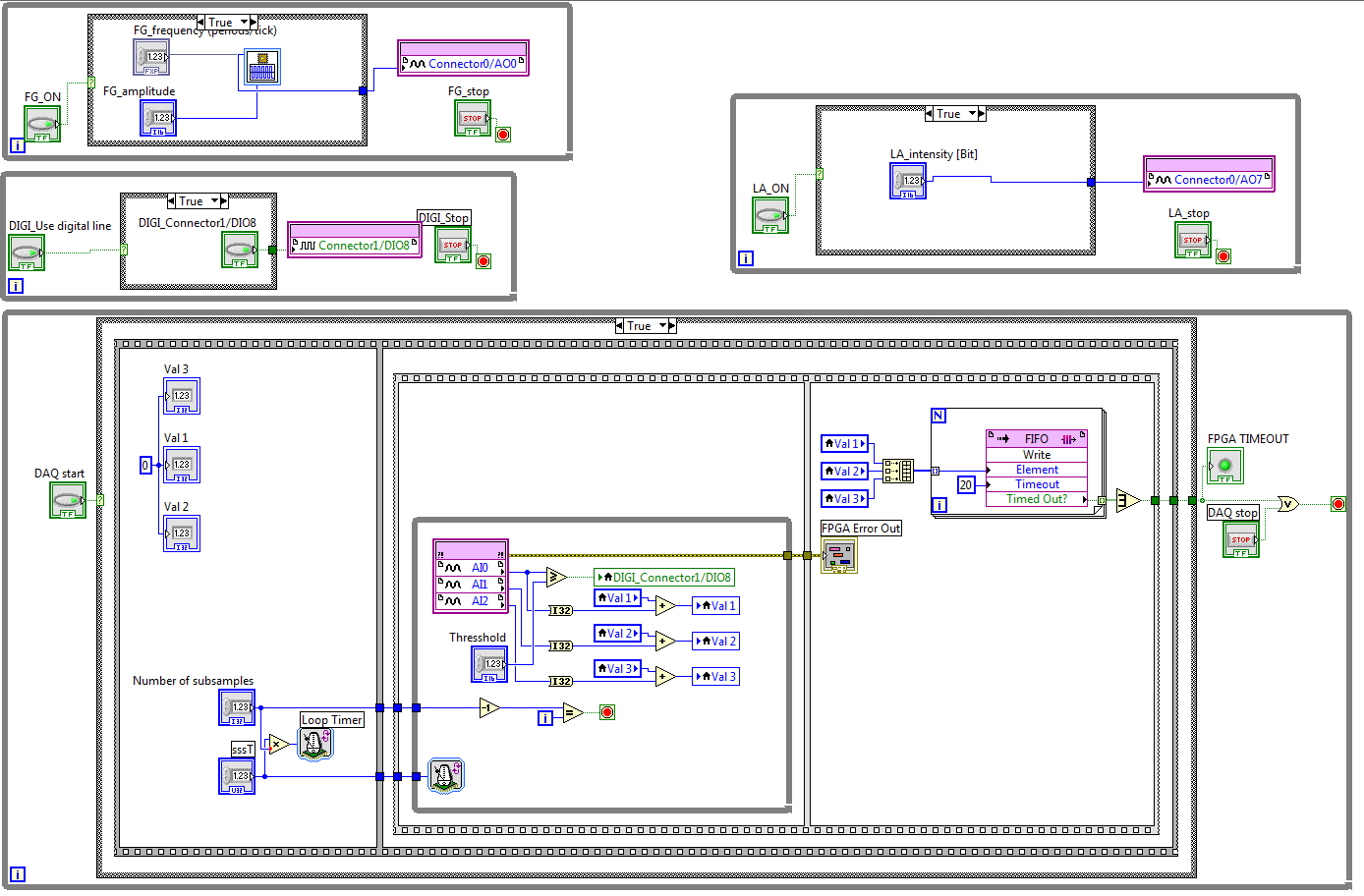

Supplement: Supplementary file 3 [file Data_Sheet_1.ZIP › NewSort/fpga.png]
